# Phosphate-to-alanine ratio and bilirubin-to-androsterone glucuronide ratio are the hub metabolites in upper gastrointestinal cancers: a Mendelian randomisation (MR) study

**DOI:** 10.3332/ecancer.2024.1731

**Published:** 2024-07-23

**Authors:** Pengkhun Nov, Duanyu Wang, Chongyang Zheng, Syphanna Sou, Socheat Touch, Samnang Kouy, Virak Vicheth, Lilin Li, Yangfeng Zhang, Xiang Liu, Changqian Wang, Peizan Ni, Qianzi Kou, Ying Li, Arzoo Prasai, Wen Fu, Wandan Li, Kunpeng Du, Jiqiang Li

**Affiliations:** 1 Department of Radiation Oncology, Oncology Center, Zhujiang Hospital of Southern Medical University, Guangzhou, Guangdong Province 510282, China; 2 Department of Radiation Oncology and Oncology, Khmer-Soviet Friendship Hospital of University of Health Sciences, Phnom Penh 120110, Cambodia; a https://orcid.org/0000-0002-0684-7291; b https://orcid.org/0000-0002-585-5911; † These authors contributed equally to this work.

**Keywords:** esophageal cancer, gastric cancer, metabolites, mendelian randomisation (MR), genome wide association study (GWAS)

## Abstract

**Objective:**

Upper gastrointestinal (UGI) cancers, particularly esophageal cancer (EC) and gastric cancer (GC) represent a significant health burden with complex etiologies. Metabolic alterations are known to play a crucial role in cancer development and progression. Identifying key metabolic biomarkers may offer insights into the pathophysiology of UGI cancers and potential therapeutic targets. This study aimed to investigate the causal associations between 1,400 types of metabolites, specifically phosphate-to-alanine and bilirubin-to-androsterone glucuronide, and the risk of developing UGI cancers using Mendelian randomisation (MR) analysis.

**Method:**

We conducted a two-sample MR study utilising genetic instruments identified from large-scale genome-wide association studies (GWASs) for metabolic traits. The outcomes were derived from GWAS datasets of UGI cancer patients, including EC and GC. Several MR methods were employed to ensure the robustness of the findings, including inverse variance weighted (IVW), MR-Egger and weighted median approaches.

**Results:**

Our analysis found a total of 44 metabolites associated with EC and 15 metabolites associated with GC. The MR analyses revealed a significant causal relationship between the phosphate-to-alanine ratio (EC: OR = 1.002,95% CI = 1.00034−1.0037, *p* = 0.0037; GC: OR = 1.24,95% CI = 1.046−1.476, *p* = 0.01) and increased risk of UGI cancers. In contrast, the bilirubin-to-androsterone glucuronide ratio (EC: OR = 0.998,95% CI = 0.997−0.999, *p* = 0.03; GC: OR = 0.80,95% CI = 0.656−0.991, *p* = 0.04) was inversely associated with the risk, suggesting a potential protective effect.

**Conclusion:**

Our findings suggest that the phosphate-to-alanine ratio and bilirubin-to-androsterone glucuronide ratio are key hub metabolites in the etiology of UGI cancers. These metabolic ratios could serve as potential biomarkers for early detection or targets for therapeutic intervention. Further research is warranted to elucidate the underlying biological mechanisms and to validate the clinical utility of these associations.

## Introduction

Upper gastrointestinal (UGI) cancers, encompassing malignancies of the esophagus and stomach, remain a leading cause of cancer-related morbidity and mortality worldwide [1]. The prognosis in many countries remains dire, largely due to the absence of effective screening programs [2, 3]. Understanding the mechanisms underlying the initiation and progression of these cancers is crucial for developing effective prevention and treatment approaches. In recent years, there has been increasing evidence that metabolites, small molecules involved in cellular metabolism, are linked to the causal pathways of UGI cancers. Advances in molecular biology and the development of various omics approaches have significantly enhanced molecular epidemiological studies in this field [4].

An imbalance in metabolism is increasingly recognised as a crucial element in the development of UGI cancers [5]. Beyond the well-established alterations in glucose metabolism, such as the Warburg effect, disturbances in the metabolism of nucleotides, lipids and amino acids have also been reported in both lab and clinical research [6–8]. Metabolites, which are the end products of intricate interplays between inherent metabolic processes, genetic factors and environmental conditions, act as a snapshot of these biochemical processes. Metabolomics, utilising high-throughput techniques, allows for the detailed detection and quantification of a vast range of small-molecule metabolites (with molecular weights less than 1,000 Da) from a single sample. This approach is instrumental in discovering new biomarkers and in deepening our understanding of the mechanisms of cancer development [9, 10]. Moreover, it paves the way for uncovering novel preventative strategies and therapeutic targets [11].

Previous observational studies have indicated a link between metabolites and an increased likelihood of developing UGI cancer [4, 12–15]. However, observational studies are susceptible to biases and confounding factors, limiting their ability to establish causality. Mendelian randomisation (MR) was initially conceptualised and developed as a method to provide robust evidence of causality through the analysis of genetic variation in relation to exposure and outcome, serving as a potential alternative to randomised controlled trials [16]. This approach leverages the random allocation of genetic variation at conception, well before the onset of disease, making MR a valuable tool for establishing causality and mitigating the risk of reverse causality, independent of confounders typically present in study designs [17]. Here, we utilised MR to investigate the histophysiology and pathophysiological involvement of the metabolites in the development of digestive tract cancers, achieved by a recent statistical summary from a genome-wide association study (GWAS) focused on metabolites [18].

The phosphate-to-alanine ratio and the bilirubin-to-androsterone glucuronide ratio have been implicated in various metabolic processes relevant to cancer biology. Phosphate is essential for energy metabolism and cellular signaling, while alanine, a non-essential amino acid, can be utilised by cancer cells to support gluconeogenesis and energy production under hypoxic conditions [33, 41]. Bilirubin, a product of heme catabolism, exhibits antioxidant properties, whereas androsterone glucuronide, a metabolite of steroid hormones, may influence cancer growth through hormonal pathways [60, 63]. This MR study aims to systematically investigate the causal relationships between 1,400 types of metabolites, specifically phosphate-to-alanine and bilirubin-to-androsterone glucuronide, and the risk of developing UGI cancers. By doing so, we seek to elucidate the potential of these ratios as hub metabolites in the metabolic landscape of UGI cancers and to explore their utility as biomarkers for cancer risk or progression. The findings could provide a foundation for novel preventive and therapeutic strategies targeting metabolic dysregulation in UGI malignancies.

## Materials and methods

### Study design

**Figure 1.**
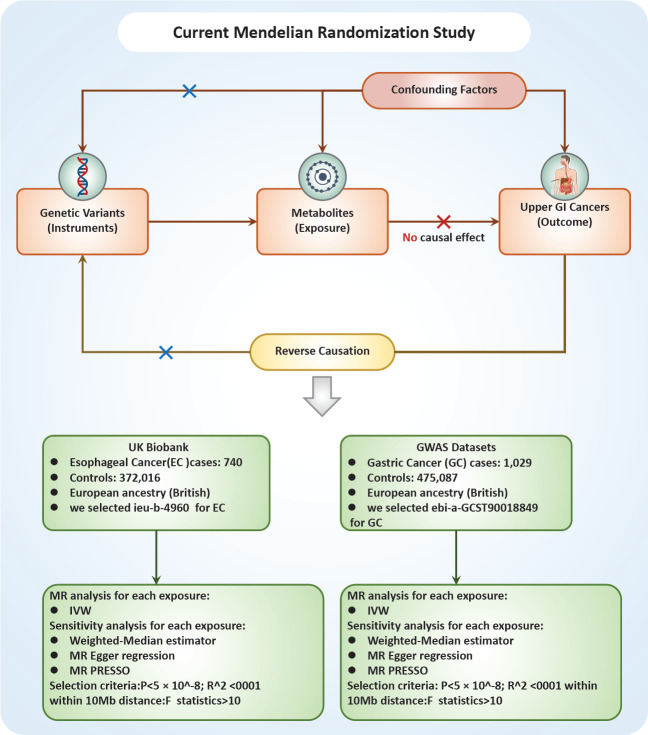
Flowchart of the study design.

The relationship between a vast array of metabolites and UGI cancers was examined in this study through the application of two-sample MR analyses. MR exploits genetic variants as surrogates for modifiable risk factors to infer causality. For MR to provide trustworthy conclusions about causal relationships, it is imperative that the instrumental variables (IVs) employed meet three critical criteria: First, the genetic variant must have a direct association with the metabolite of interest (the exposure). Second, the genetic variant should not be associated with confounding factors that could influence both the exposure and the outcome, ensuring that observed associations are not spurious. Finally, the genetic variant should affect the outcome solely through its impact on the exposure, with no alternative pathways influencing the outcome Figure 1.

### Data sources for exposure and outcome

The sources of metabolite-wide GWAS data. The statistics summary of GWAS for each metabolite is publicly available from the European GWASs (accession number: GCST90199621-90201020) or non-European GWASs (accession number: GCST90201021-90204063) [19]. To identify relevant data for each cancer type, we used cancer-specific keywords to search (https://gwas.mrcieu.ac.uk/). For esophageal cancer (EC), we selected ieu-b-4960 (EC), and for gastric cancer (GC), we chose ebi-a-GCST90018849 (GC). Following this, we downloaded the corresponding data (from https://www.ebi.ac.uk/gwas/) based on the IDs of each cancer type, which was used for the analysis of the relation between 1,400 types of metabolites and UGI cancers. A reference panel derived from Sardinian sequences was utilised [20] to estimate approximately 22 million single-nucleotide polymorphisms (SNPs) genotyped using high-density arrays, and correlations were assessed following adjustment for covariates. The GWAS database is a comprehensive collection of genetic variation and its association with various traits or diseases. It provides a valuable resource for researchers and clinicians interested in understanding the genetic basis of complex traits and diseases. Based on the ID of each cancer, we used online data from GWAS including 372,756 European individuals (*n* = 740 case patients and 372,016 control participants) for EC, and 476,116 European individuals (*n* = 1,029 case patients and 475,087 control participants) for GC to analyse the relationship between 1,400 type metabolites and each cancer according to IDs (https://www.ebi.ac.uk/gwas/).

### Instrument selection

In light of the large number of SNPs reaching genome-wide significance (*p* < 5 × 10^-8^) for metabolite traits, we adopted more stringent criteria (*p* < 5 × 10^-9^) for selecting genetic IVs [21]. These IVs were pinpointed by categorising them based on the linkage disequilibrium reference panel from the 1,000 Genomes Project, applying a cutoff of *R*^2^ < 0.001 at a distance of up to 1,000 kilobases (kb). Due to the comparatively smaller size of GWAS datasets for metabolites, we utilised a *p*-value threshold of 5 × 10^-8^ and a more relaxed clustering threshold (*R*^2^ < 0.1 at a distance of 500 kb) [22]. To ensure the strength of our genetic instruments, we only selected IVs with *F*-statistics greater than 10, thereby qualifying them as robust instruments for our analyses. These IVs were then extracted from the summary data for UGI cancer outcomes. We excluded any SNPs that demonstrated potential pleiotropic effects (*p* < 10^-5^) on UGI cancer, which is consistent with protocols from prior studies [23]. We harmonised the SNPs across the datasets for exposures and outcomes to ensure coherent effect size estimations for the same alleles. SNPs with effect allele frequencies greater than 0.42 or those that were incompatible with harmonisation, were omitted from our analysis [22]. This process of careful SNP selection and harmonisation ensures the integrity and consistency of our MR analysis.

### Statistical analysis

R 4.3.1 software (http://www.Rproject.org) was used to perform the analysis. Three methods were primarily utilised to reveal the causal relationship between 1,400 types of metabolites and UGI cancers: inverse variance weighting (IVW) [24], median-based weighting [25] and pattern-based weighting [26]. These analysis were primarily carried out using the software package of ‘TwoSampleMR’ (version 0.4.3) [27]. To assess heterogeneity among the selected IVs, Cochran's *Q* statistical along with corresponding values were utilised. Where the null hypothesis was rejected, we opted for random effects IVW in lieu of fixed effects [24]. In addressing the potential impact of horizontal pleiotropy, we employed the MR-Egger method. This method is particularly useful for detecting the presence of horizontal pleiotropy, indicated by a statistically significant intercept term [24]. Furthermore, we used the MR Pleiotropy Residual Sum and Outlier method, a robust approach to identify and eliminate potential horizontal pleiotropic outliers that might significantly influence our estimation results [28]. To further validate our findings, scatterplots and funnel plots were used. These plots confirmed that our results were not biased by outliers and demonstrated that the correlations observed were robust and exhibited no significant heterogeneity.

## Results

**Figure 2.**
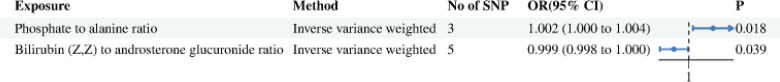
Causal estimation between metabolites and EC.

**Figure 3.**
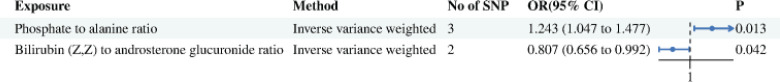
Causal estimation between metabolites and GC.

To assess the causal impact of various metabolites on EC and GC, we employed a two-sample MR analysis using the method of IVW. In our study, we found a total of 44 metabolites associated with EC and 15 metabolites associated with GC (Tables 1 and 2). Phosphate-to-alanine ratio (EC: OR = 1.002,95% CI = 1.00034− 1.0037, *p* = 0.0037; GC: OR = 1.24,95% CI = 1.046− 1.476, *p* = 0.01) and bilirubin (Z,Z) to androsterone glucuronide (EC: OR = 0.998,95% CI = 0.997− 0.999, *p* = 0.03; GC: OR = 0.80,95% CI = 0.656− 0.991, *p* = 0.04) are common metabolites of EC and GC. Among them, phosphate-to-alanine ratio are risk factor for GC and EC, while bilirubin (Z,Z) to androsterone glucuronide are protective factors for GC and EC. Our findings are summarised in Figures 2 and 3. However, neither the MR-Egger intercept test nor Cochran's *Q* test revealed pleiotropy and heterogeneity (Supplementary Tables 1 and 2).

## Discussion

MR has become a pivotal tool for demonstrating potential causal risk factors in diseases. In the present study, we used MR to establish an inverse causal relationship between metabolites and UGI cancers.

The nexus between metabolites and cancer has garnered significant attention recently. The development and progression of cancer are intrinsically linked to cellular metabolism. Cancer cells often exhibit unique metabolic pathways, such as relying on glycolysis and lactic acid fermentation for glucose metabolism instead of mitochondrial pathways [29]. Additionally, tumours may reprogram lipid metabolism, enhancing lipid uptake and accumulation, which alters the tumour microenvironment, suppresses immune responses and promotes tumour progression [30]. The complexity of tumour metabolism and the heterogeneous role of metabolites in cancer make this a challenging area of study. Metabolites are not only predictors of cancer risk but also play crucial roles in cancer treatment, where reprogramming metabolic pathways can help overcome chemotherapy resistance [31, 32]. In our MR analysis, we investigated the associations between serum metabolites and the risks of EC and GC. Our findings reveal distinct metabolite profiles associated with these cancers in the European population, as well as some common metabolites. This has led to the identification of novel candidate metabolites that may influence the risk of EC and GC. These insights offer new avenues for the treatment and management of these cancers, contributing significantly to our understanding of their etiology and mechanisms. In our investigation into EC cancer, we identified 44 metabolites related to the disease, while in GC cancer, we identified 15 related metabolites. Among them, phosphate-to-alanine ratio and bilirubin (Z, Z) to androsterone glucuronide ratio were found to be jointly connected to the risk of UGI cancers. Our findings suggest that a high phosphate-to-alanine ratio is linked to an increased risk of UGI cancers, whereas a higher bilirubin (Z, Z) to androsterone glucuronide ratio might act as a protective factor. Phosphate plays an important role in cell growth. For example, liver regeneration requires a large amount of phosphate to synthesize nucleotide triphosphate due to DNA replication. After hepatectomy, a decrease in phosphate indicates liver cell regeneration and a lack or delay of this decrease suggests impaired regeneration [33]. In small-cell lung cancer, diminished serum phosphate has been associated with an abnormal increase in fibroblast growth factor 23 [34]. Moreover, phosphates like nicotinamide adenine dinucleotide are central to maintaining redox homeostasis in cancer [35]. For instance, in gliomas with functional phosphate and tensin homologs, hypoxia induces endogenous production of cytoplasmic reactive oxygen species (ROS) and tumour cell growth by activating nicotinamide adenine dinucleotide phosphate (NADPH) oxidase, contributing to radiation resistance [36]. Lung cancer cells typically exhibit elevated ROS and NADPH levels [37], and NADPH is also associated with pancreatic cancer risk [38]. Research by Shi *et al* [39] indicated that Myoferlin disrupts the redox balance, promotes ROS production and increases the ratio of NADPH/NADP+, thereby accelerating the metastasis of GC. These studies corroborate our findings that elevated phosphate levels, possibly through increased NADPH production, lead to enhanced ROS production, promoting tumour cell proliferation and metastasis in UGI cancers. A critical aspect of cancer cell interaction with their environment involves the exchange of metabolites, especially amino acids [40]. A significant portion of metabolic alterations in cancer relates to amino acid metabolism and biosynthesis. Alanine is a non-essential amino acid primarily synthesized in the mitochondrial matrix. Alanine aminotransferase competes with pyruvate dehydrogenase (PDH), and PDH oxidizes decarboxylation to form acetyl CoA. PDH is responsible for the oxidative decarboxylation of pyruvate to form acetyl CoA, a process that is subdued under hypoxic conditions, leading to increased alanine synthesis [41]. In hepatocellular carcinoma, the alanine metabolism pathway undergoes significant changes typically marked by a reduction in alanine content [42]. Interestingly, in breast cancer, β- Alanine and alanine have the same chemical formula but differ in structure. The increase in their content due to a decrease of 4-aminobutyrate aminotransferase normally catalyzes β- Alanine into malonic acid semialdehyde, which can be converted into acetyl coenzyme A by simultaneously reducing NAD+to NADH [43]. Furthermore, the synthesis of alanine in breast cancer is influenced by the α-ketoglutarate demand, driven by pyruvate uptake in the tumour microenvironment [44]. In our study, we also observed a correlation between the high risk of UGI cancers and reduced alanine levels, which is consistent with the findings in hepatocellular carcinoma. This suggests that the metabolic pathway of alanine in gastrointestinal tumours might follow a similar pattern, with cancer cells potentially absorbing and deaminating alanine to form pyruvate, an essential carbon source for synthesizing other compounds. Thus, our results indicate that the phosphate-to-alanine ratio is linked to the increased risk of UGI cancers. The interaction between phosphate and alanine and the impact of their relative concentrations could serve as a potential predictive biomarker for UGI cancers. Bilirubin, the end product of red blood cell degradation, is categorised into direct bilirubin (DBIL) and indirect bilirubin (IBIL). Previous studies have highlighted the antioxidant, anti-inflammatory and anticancer properties of bilirubin, especially IBIL [45–47]. In addition, studies have confirmed that high bilirubin is associated with favourable prognosis and lower incidence rates in other cancers, including lung, breast and colorectal cancer [48–50]. However, the study by Wei *et al* [51] found reduced levels of total bilirubin (TBIL), DBIL and IBIL in GC patients, leaving the causal relationship between bilirubin reduction and GC unclear. In esophageal squamous cell carcinoma, research by Huang *et al* [52] suggested that elevated preoperative serum bilirubin levels (including non-conjugated bilirubin, conjugated bilirubin and TBIL) have been linked to a longer overall OS. Typically, elevated serum bilirubin is an indicator of liver dysfunction [53]. Given the liver’s involvement in multiple metabolic processes, liver cancer patients often experience abnormalities in multiple liver function indicators in the late stage. Therefore, composite index ratios like the albumin bilirubin score (ALBI) have been increasingly used to evaluate liver cancer prognosis, demonstrating substantial predictive value [54]. ALBI has also been recognised as a prognostic factor for GC, EC, colon cancer, pancreatic cancer and non-small cell lung cancer [55–58]. A retrospective study involving 628 patients undergoing radical gastrectomy for GC showed that those with higher preoperative ALBI levels experienced a significantly higher incidence of postoperative complications [59]. Similarly, in patients with ampullary adenocarcinoma undergoing radical pancreaticoduodenectomy, a high preoperative TBIL to albumin ratio was identified as an independent protective factor against recurrence [60]. In EC, a higher incidence of postoperative anastomotic leakage was observed to be 46.3% in the high ALBI group versus 27.5% in the low ALBI group (*p* = 0.038) [58].

Steroid hormone metabolism typically activates pathways involved in cell proliferation, survival, migration and invasiveness and is related to cancer initiation and progression closely [61]. Androsterone glucuronide, a liver metabolite of a glucosyltransferase-modified steroid oestrogen and testosterone, as well as a metabolite of the steroid dihydrotestosterone found in serum, exhibits weaker androgen activity. A case-control study on ovarian cancer has revealed that elevated androsterone glucuronide levels lead to an increased risk of non-serious ovarian cancer in women [62]. Similarly, another study conducted by Kalogera *et al* [61, 63] also found significantly increased androsterone glucuronide levels in patients with lobular neoplasia compared to those with benign breast disease. Thus, androsterone glucuronide could be a valuable marker for assessing the efficacy of endocrine therapy for breast cancer by detecting its concentration in the blood and evaluating total androgen activity in an accurate way [63]. Some studies have shown a weak but positive correlation between the levels of androsterone glucuronide and the risk of prostate cancer [61]. Interestingly, Chinese men have lower plasm levels of androsterone glucuronide compared to Caucasian males in Western countries [64]. These studies suggest a positive correlation between androsterone glucuronide and the risk of hormone-dependent malignant tumours. However, the relationship between androsterone glucuronide and the risk of non-hormone-dependent malignant tumours remains unclear. In our study, we found that the increase in bilirubin (Z, Z) to androsterone glucuronide ratio is a protective factor for UGI cancers. This protective effect is primarily attributed to the anticancer effect of bilirubin. Given the uncertain relationship between androsterone glucuronide and non-hormone-dependent malignant tumours, it is possible that androsterone glucuronide contributes to the risk of EC and GC. However, bilirubin's anticancer effects likely outweigh any potential risks posed by androsterone glucuronide. The synergistic effect of bilirubin’s anticancer properties and the potential protective role of androsterone glucuronide suggests that an increased bilirubin (Z, Z) to androsterone glucuronide ratio may improve the prognosis of EC and GC. Further research is necessary to elucidate the specific underlying mechanisms.

## Limitations

This study is subject to several limitations. First, the metabolomic profiles were derived from non-fasting plasma samples. Although corrections were made considering the time since last eating or drinking, residual variances that have not been accounted for could remain. Second, the investigation concentrated on gene-metabolite associations that are currently supported by gene expression data and biological knowledge, with a particular emphasis on those involving effector genes. This approach may overlook the potential significance of other metabolites or metabolic ratios that, despite their strong heritability, were not the primary focus of this study. To uncover the effector genes corresponding to these additional metabolites and ratios, future studies will need to integrate more comprehensive gene expression data and a deeper understanding of metabolic pathways. Third, our study's implementation of MR was limited by the fact that the majority of metabolites and their corresponding ratios were linked to only one IV. This restriction prevented us from using several sensitivity analyses, such as MR-Egger regression, which requires multiple IVs for robustness checks against potential biases like horizontal pleiotropy. Nevertheless, we sought to minimize the impact of horizontal pleiotropy by selecting IVs that are closely associated with specific effector genes known to directly influence metabolite concentrations. We also manually examined instances of metabolic pleiotropy and excluded IVs that affected multiple metabolites not part of the same biological pathway. Although these measures aimed to curtail bias, we recognize that it cannot be entirely ruled out due to current limitations in metabolomic profiling and incomplete data on metabolite-protein interactions. Future studies with a more exhaustive analysis of the metabolome will be vital for a more precise delineation of genetic impacts on metabolites.

Moreover, the demographic scope of this study was largely confined to older adults of European ancestry. Broadening the research to include a wider range of ages and ethnic backgrounds will be instrumental in validating and extending the applicability of our findings regarding genetic variations in metabolites and their ratios across different populations. Furthermore, our study lacks SNP, using a single SNP as an instrument in MR studies can lead to weak instrument bias, where the instrument is not sufficiently associated with the exposure, leading to imprecise estimates and potentially biased results.

## Conclusion

In this MR study, we scrutinized the causal links between metabolite ratios and the risk of UGI cancers. Our rigorous analysis identified two critical metabolic ratios, the phosphate-to-alanine ratio and the bilirubin-to-androsterone glucuronide ratio, as significant players in the context of UGI cancers. The results suggest a potential risk relationship with an increased phosphate-to-alanine ratio, hinting at disruptions in energy metabolism and amino acid balance that may contribute to cancer development. Conversely, a higher bilirubin-to-androsterone glucuronide ratio appears to be associated with a reduced risk of UGI cancers, possibly reflecting the protective antioxidative effects of bilirubin and the influence of steroid hormone metabolism. These findings provide a promising step forward in understanding the metabolic underpinnings of UGI cancers and highlight the utility of MR in uncovering potential biomarkers for disease risk. While these results are encouraging, further investigation is essential to confirm these relationships and to explore their clinical implications. Ultimately, understanding these hub metabolites could lead to improved strategies for prevention, early detection and targeted treatment of UGI cancers.

## Conflicts of interest

The authors declare no competing interests.

## Funding

This study was supported by the Natural Science Foundation of Guangdong Province (Grant No. 2023A1515012548).

## Consent for publication

All the authors of the article agreed to be published in the journal.

## Ethics approval

Not applicable.

## Availablity of data and material

All the data for this article can be found on GWAS database and UKbiobank database.

## Author contributions

Pengkhun Nov collect data, analysing, interpretation of data, Duanyu Wang drafting the article; Wandan Li choose the topic; Kunpeng Du and Jiqiang Li designing, revising and guiding the study. The authors read and approved.

## Figures and Tables

**Table 1.** Causal estimation between metabolites and EC.

Exposure	Method	nsnp	pval	or	or_lci95	or_uci95
Gentisate levels	IVW	4	0.038586	1.001446	1.000076	1.002819
Hexanoylcarnitine levels (Biocrates platform)	IVW	3	0.027575	1.000552	1.000061	1.001044
Homocitrulline levels	IVW	2	0.028067	1.002321	1.00025	1.004398
Propionylglycine levels	IVW	2	0.013841	0.997617	0.995724	0.999514
Octanoylcarnitine (c8) levels	IVW	3	0.049033	1.000545	1.000002	1.001088
Gamma-glutamylmethionine levels	IVW	5	0.014381	0.998393	0.997108	0.99968
N2,n2-dimethylguanosine levels	IVW	2	0.014332	1.002466	1.000492	1.004444
Hexanoylglycine levels	IVW	2	0.021041	1.000855	1.000129	1.001581
Dimethylarginine (sdma + adma) levels	IVW	2	0.04918	0.998269	0.996547	0.999994
Hexanoylglutamine levels	IVW	5	0.026742	1.000773	1.000089	1.001458
Cis-4-decenoylcarnitine (C10:1) levels	IVW	4	0.045316	1.000542	1.000011	1.001072
Nonanoylcarnitine (C9) levels	IVW	3	0.039281	1.000582	1.000029	1.001136
1-palmitoyl-2-arachidonoyl-GPI (16:0/20:4) levels	IVW	2	0.027394	1.001753	1.000195	1.003312
Nisinate (24:6n3) levels	IVW	3	0.008633	1.001742	1.000442	1.003045
Dihomo-linolenoylcarnitine (C20:3n3 or 6) levels	IVW	2	0.002193	0.997049	0.995165	0.998936
Arachidonoylcarnitine (C20:4) levels	IVW	3	0.001527	0.997315	0.995658	0.998975
3-hydroxybutyroylglycine levels	IVW	4	0.016788	0.998206	0.996738	0.999676
N-acetyl-isoputreanine levels	IVW	4	0.031588	0.998479	0.997094	0.999866
Methyl vanillate sulfate levels	IVW	2	0.016476	1.002051	1.000374	1.003731
4-methylhexanoylglutamine levels	IVW	6	0.023752	1.000855	1.000114	1.001597
Branched-chain, straight-chain, or cyclopropyl 12:1 fatty acid levels	IVW	2	0.0229	1.00252	1.000349	1.004696
Ceramide (d18:1/16:0) levels	IVW	3	0.040343	1.001651	1.000073	1.003231
Trans-urocanate levels	IVW	2	0.005697	1.0029	1.000843	1.00496
Caproate (6:0) levels	IVW	2	0.035751	1.001099	1.000073	1.002125
Nonadecanoate (19:0) levels	IVW	2	0.00211	1.003191	1.001155	1.005231
X-18886 levels	IVW	2	0.010824	0.997854	0.996207	0.999504
X-21733 levels	IVW	2	0.000775	1.003113	1.001297	1.004933
X-24418 levels	IVW	5	0.035589	1.001148	1.000077	1.00222
X-25957 levels	IVW	2	0.031283	1.001968	1.000177	1.003762
X-25519 levels	IVW	2	0.031996	1.002132	1.000183	1.004084
N-acetyltyrosine levels	IVW	4	0.039919	0.999656	0.999328	0.999984
Alpha-ketoglutarate to succinate ratio	IVW	2	0.028957	0.997751	0.995737	0.999769
Phosphate to alanine ratio	IVW	3	0.018051	1.002041	1.000349	1.003735
Phosphate to phosphoethanolamine ratio	IVW	3	0.027151	0.998304	0.996802	0.999808
Oleoyl-linoleoyl-glycerol (18:1 to 18:2) [2] to linoleoyl-arachidonoyl-glycerol (18:2 to 20:4) [2] ratio	IVW	2	0.024901	1.002207	1.000278	1.004139
Glycine to phosphate ratio	IVW	2	0.019122	0.997637	0.995664	0.999613
Adenosine 5′-monophosphate (AMP) to citrate ratio	IVW	2	0.035565	0.997906	0.995958	0.999859
Adenosine 5′-monophosphate (AMP) to isoleucine ratio	IVW	4	0.010368	0.998188	0.996805	0.999573
Adenosine 5′-monophosphate (AMP) to valine ratio	IVW	3	0.041684	0.998147	0.996367	0.99993
Cysteinylglycine to taurine ratio	IVW	2	0.018044	0.997386	0.995225	0.999552
Cortisone to 4-cholesten-3-one ratio	IVW	3	0.026977	1.001789	1.000204	1.003376
Alpha-ketoglutarate to trans-4-hydroxyproline ratio	IVW	4	0.035469	1.001519	1.000103	1.002937
Phosphate to EDTA ratio	IVW	3	0.033036	1.001788	1.000144	1.003436
Bilirubin (Z,Z) to androsterone glucuronide ratio	IVW	5	0.038551	0.998749	0.997565	0.999934

**Table 2.** Causal estimation between metabolites and GC.

Exposure	Method	nsnp	pval	or	or_lci95	or_uci95
Catechol sulfate levels	IVW	2	0.018879	0.595349	0.386149	0.917885
4-hydroxyglutamate levels	IVW	2	0.018322	0.811457	0.682149	0.965276
Gamma-CEHC glucuronide levels	IVW	2	0.001425	0.301233	0.144108	0.629677
Carnitine C14:1 levels	IVW	2	0.019502	0.358908	0.151902	0.848017
Sphingomyelin (d18:0/20:0, d16:0/22:0) levels	IVW	2	0.007016	0.455139	0.256816	0.806613
2,4-di-tert-butylphenol levels	IVW	3	0.02333	0.525574	0.301443	0.916352
X-24295 levels	IVW	2	0.020172	0.663254	0.469066	0.937834
X-24337 levels	IVW	6	0.044879	1.271046	1.005489	1.606738
Bilirubin degradation product, C17H18N2O4 (1) levels	IVW	2	0.021045	1.621617	1.075457	2.445138
Adenosine 5′-monophosphate (AMP) to phenylalanine ratio	IVW	2	0.004944	0.652417	0.484378	0.878752
Phosphate to alanine ratio	IVW	3	0.01309	1.24341	1.046835	1.476897
Uridine to pseudouridine ratio	IVW	2	0.019666	0.718572	0.544341	0.948571
Adenosine 5′-monophosphate (AMP) to tryptophan ratio	IVW	2	0.006357	0.640254	0.464796	0.881948
Alanine to asparagine ratio	IVW	2	0.024378	0.810625	0.675198	0.973214
Bilirubin (Z,Z) to androsterone glucuronide ratio	IVW	2	0.041506	0.806659	0.656101	0.991767

**Supplementary Figure 1.**
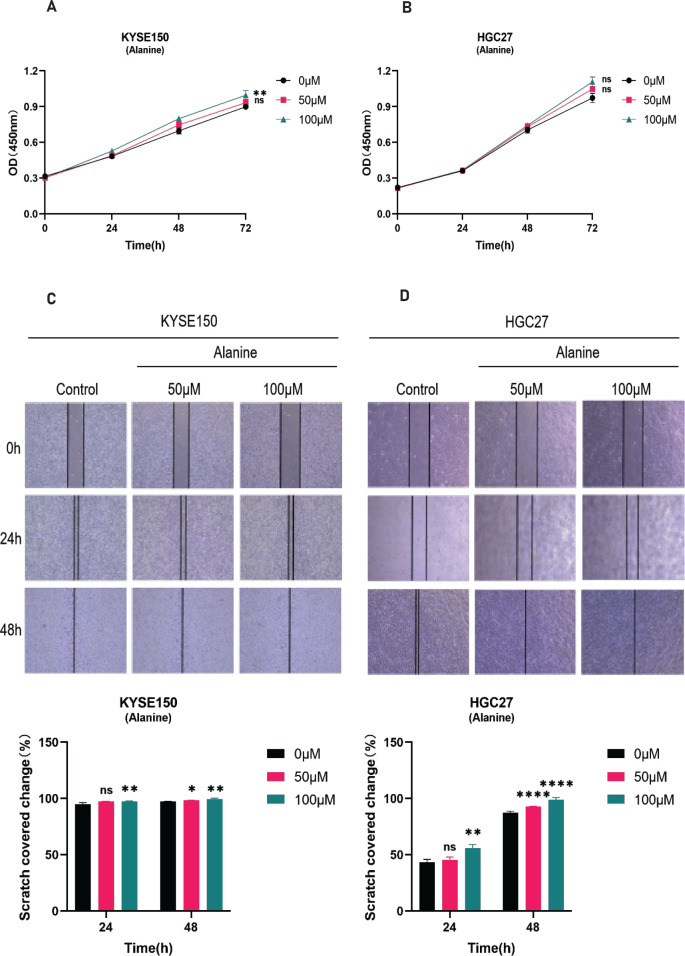
A-B: CCK-8 method was used to detect the proliferation of KYSE150 and HGC27 cells. Divide different cell lines into three groups and treat the cells with different concentrations of alanine (0, 50, 100 μ M). Measure the absorbance values of cells at different time points (0, 24, 48, 72 hours) using the CCK-8 method, and evaluate the proliferation of cells through plotting. C-D: Scratch assay for detecting the migration of KYSE150 and HGC27 cells. KYSE150 and HGC27 cells were treated with different concentrations of alanine for 24 and 48 hours, and the scratch area at different time points was calculated to evaluate the migration ability of the cells. All experiments were repeated 3 times, and the results were expressed as mean ± SDs. Compared with the control group, there was no significant difference in ns, with * * *p *< 0.05, *p* < 0.01, * * * *p* < 0.005, * * * * < 0.001

## Supplementary material

**Supplementary Table 1.** The pleiotropy of causal relationship between all metabolites and esophageal cancer.

Exposure	Egger_intercept	se	pval
Gentisate levels	1.24E-05	0.000176497	0.9503632
Hexanoylcarnitine levels (Biocrates platform)	3.70E-05	0.000250697	0.90679916
Homocitrulline levels			
Propionylglycine levels			
Octanoylcarnitine (c8) levels	−4.97E-05	0.000229692	0.864228296
Gamma-glutamylglycine levels			
N2,n2-dimethylguanosine levels			
Hexanoylglycine levels			
Dimethylarginine (sdma + adma) levels			
Hexanoylglutamine levels	−2.42E-05	0.000167001	0.894008433
Cis-4-decenoylcarnitine (C10:1) levels	−8.11E-06	0.000124949	0.95415757
Nonanoylcarnitine (C9) levels	−9.74E-06	0.000238869	0.974057642
1-palmitoyl-2-arachidonoyl-GPE (16:0/20:4) levels	0.000102248	0.000216733	0.719371536
Nisinate (24:6n3) levels	−0.000114913	0.000496313	0.855153197
Dihomo-linolenoylcarnitine (C20:3n3 or 6) levels			
Arachidonoylcarnitine (C20:4) levels	−0.000170963	0.00021786	0.576415525
3-hydroxyoleoylcarnitine levels	0.000352436	0.000286707	0.434759346
N-acetyl-isoputreanine levels	7.34E-05	0.000235257	0.784515943
Methyl vanillate sulfate levels			
4-methylhexanoylglutamine levels	−0.000210567	0.000163812	0.268020447
Branched-chain, straight-chain, or cyclopropyl 12:1 fatty acid levels			
Ceramide (d18:1/16:0) levels	4.28E-05	0.000186666	0.856671636
Trans-urocanate levels			
Caproate (6:0) levels			
Nonadecanoate (19:0) levels			
X-18886 levels			
X-21733 levels			
X-24418 levels	−0.000237333	0.000518479	0.678251784
X-25957 levels			
N-acetyltyrosine levels	−2.69E-05	0.000151417	0.875556823
Alpha-ketoglutarate to succinate ratio			
Phosphate to alanine ratio	0.000176286	0.000228167	0.581219102
Phosphate to phosphoethanolamine ratio	−0.000366552	0.000272115	0.406542922
Oleoyl-linoleoyl-glycerol (18:1 to 18:2) [2] to linoleoyl-arachidonoyl-glycerol (18:2 to 20:4) [2] ratio			
Glycine to phosphate ratio			
Adenosine 5′-monophosphate (AMP) to citrate ratio			
Adenosine 5′-monophosphate (AMP) to isoleucine ratio	−0.000279944	0.000237009	0.35896969
Adenosine 5′-monophosphate (AMP) to valine ratio	−0.000279482	0.000235303	0.445499084
Cysteinylglycine to taurine ratio			
Cortisone to 4-cholesten-3-one ratio	−0.00022983	0.000323003	0.606295739
Alpha-ketoglutarate to trans-4-hydroxyproline ratio	0.000150662	0.000198571	0.527237598
Phosphate to EDTA ratio	0.000227554	0.000217679	0.485882116
Bilirubin (Z,Z) to androsterone glucuronide ratio	−6.72E-05	0.000171369	0.721325827

**Supplementary Table 2.** The heterogeneity of causal relationship between all metabolites and esophageal cancer.

Exposure	Method	Q	Q_df	Q_pval
Gentisate levels	IVW	1.414904	3	0.702045
Hexanoylcarnitine levels (Biocrates platform)	IVW	1.029527	2	0.597642
Propionylglycine levels	IVW	0.010371	1	0.918884
Octanoylcarnitine (c8) levels	IVW	0.092124	2	0.954983
Gamma-glutamylthreonine levels	IVW	1.643063	4	0.801032
N2,n2-dimethylguanosine levels	IVW	0.07995	1	0.777366
Hexanoylglycine levels	IVW	0.106043	1	0.744695
Dimethylarginine (sdma + adma) levels	IVW	0.157792	1	0.691198
Hexanoylglutamine levels	IVW	0.082255	4	0.999177
Cis-4-decenoylcarnitine (C10:1) levels	IVW	0.150189	3	0.985199
Nonanoylcarnitine (C9) levels	IVW	0.029438	2	0.985389
1-palmitoyl-2-arachidonoyl-GPE (16:0/20:4) levels	IVW	0.903521	2	0.636507
Nisinate (24:6n3) levels	IVW	0.291147	2	0.864526
Dihomo-linolenoylcarnitine (C20:3n3 or 6) levels	IVW	0.161271	1	0.687989
Arachidonoylcarnitine (C20:4) levels	IVW	0.869377	2	0.647466
3-hydroxyoleoylcarnitine levels	IVW	4.646325	2	0.097963
N-acetyl-isoputreanine levels	IVW	0.854561	3	0.836377
Methyl vanillate sulfate levels	IVW	1.391044	1	0.238229
4-methylhexanoylglutamine levels	IVW	3.63197	5	0.60352
Branched-chain, straight-chain, or cyclopropyl 12:1 fatty acid levels	IVW	0.948363	1	0.330136
Ceramide (d18:1/16:0) levels	IVW	0.157398	2	0.924318
Trans-urocanate levels	IVW	0.933185	1	0.334037
Caproate (6:0) levels	IVW	0.347583	1	0.555484
Nonadecanoate (19:0) levels	IVW	0.006784	1	0.934355
X-18886 levels	IVW	0.283655	1	0.594316
X-21733 levels	IVW	0.117327	1	0.731952
X-24418 levels	IVW	2.540923	4	0.637323
X-25957 levels	IVW	0.02632	1	0.871121
N-acetyltyrosine levels	IVW	1.578843	3	0.664197
Alpha-ketoglutarate to succinate ratio	IVW	0.070626	1	0.790428
Phosphate to alanine ratio	IVW	0.790348	2	0.673563
Phosphate to phosphoethanolamine ratio	IVW	1.823215	2	0.401878
Oleoyl-linoleoyl-glycerol (18:1 to 18:2) [2] to linoleoyl-arachidonoyl- glycerol (18:2 to 20:4) [2] ratio	IVW	0.011637	1	0.914094
Glycine to phosphate ratio	IVW	0.203197	1	0.652153
Adenosine 5′-monophosphate (AMP) to citrate ratio	IVW	0.075328	1	0.783732
Adenosine 5′-monophosphate (AMP) to isoleucine ratio	IVW	2.094438	3	0.553039
Adenosine 5′-monophosphate (AMP) to valine ratio	IVW	2.305697	2	0.315736
Cysteinylglycine to taurine ratio	IVW	0.69657	1	0.403939
Cortisone to 4-cholesten-3-one ratio	IVW	0.726605	2	0.695376
Alpha-ketoglutarate to trans-4-hydroxyproline ratio	IVW	2.415661	3	0.490726
Phosphate to EDTA ratio	IVW	1.149102	2	0.562958
Bilirubin (Z,Z) to androsterone glucuronide ratio	IVW	2.28896	4	0.68278

**Supplementary Table 3.** The pleiotropy of causal relationship between all metabolites and gastric cancer.

Exposure	Egger_intercept	se	pval
Catechol sulfate levels			
4-hydroxyglutamate levels			
Gamma-CEHC glucuronide levels			
Carnitine C14:1 levels			
Sphingomyelin (d18:1/20:1, d18:2/20:0) levels			
2,4-di-tert-butylphenol levels	−0.105602428	0.264966966	0.758558613
X-24295 levels			
X-24337 levels	−0.020014927	0.03841537	0.62987063
Bilirubin degradation product, C17H18N2O4 (1) levels			
Adenosine 5′-monophosphate (AMP) to phenylalanine ratio			
Phosphate to alanine ratio	0.013133583	0.018398392	0.605323144
Uridine to pseudouridine ratio			
Adenosine 5′-monophosphate (AMP) to tryptophan ratio			
Alanine to asparagine ratio			
Bilirubin (Z,Z) to androsterone glucuronide ratio			

**Supplementary Table 4.** The heterogeneity of causal relationship between all metabolites and gastric cancer.

Exposure	Method	Q	Q_df	Q_pval
Catechol sulfate levels	IVW	1.064121	1	0.302277
4-hydroxyglutamate levels	IVW	0.00116	1	0.97283
Gamma-CEHC glucuronide levels	IVW	0.275868	1	0.599422
Carnitine C14:1 levels	IVW	0.066075	1	0.79714
Sphingomyelin (d18:0/20:0, d16:0/22:0) levels	IVW	0.234892	1	0.62792
2,4-di-tert-butylphenol levels	IVW	0.386574	2	0.824245
X-24295 levels	IVW	0.161678	1	0.687616
X-24337 levels	IVW	0.644154	5	0.985893
Bilirubin degradation product, C17H18N2O4 (1) levels	IVW	0.081743	1	0.77495
Phosphate to alanine ratio	IVW	0.52519	2	0.769053
Uridine to pseudouridine ratio	IVW	0.634094	1	0.425858
Adenosine 5′-monophosphate (AMP) to tryptophan ratio	IVW	0.108693	1	0.741637
Alanine to asparagine ratio	IVW	0.44321	1	0.505577
Bilirubin (Z,Z) to androsterone glucuronide ratio	IVW	0.321286	1	0.570836
